# RNA interference in crop protection: opportunities and challenges during the transition to commercialization

**DOI:** 10.1002/ps.70806

**Published:** 2026-04-21

**Authors:** Elisabetta Sergi, Anna Narduzzo, Alessandra Di Canito, Francesco Favaretto, Luca Nerva, Walter Chitarra, Lucia Bologna, Jacopo Bacenetti, Giuliana Maddalena, Simona Masiero, Ileana Vigentini, Gabriella De Lorenzis, Silvia Laura Toffolatti

**Affiliations:** ^1^ Department of Agricultural and Environmental Sciences (DiSAA) University of Milan Milan Italy; ^2^ Council for Agricultural Research and Economics – Research Centre for Viticulture and Enology Conegliano Italy; ^3^ Department of Agronomy, Food, Natural Resources, Animals and Environment, Agripolis University of Padua Legnaro Italy; ^4^ Department of Biomedical Surgical and Dental Sciences, University of Milan Milan Italy; ^5^ Department of Environmental Sciences and Policy (ESP) University of Milan Milan Italy; ^6^ Department of Biosciences University of Milan Milan Italy

**Keywords:** agricultural biotechnology, disease control, HIGS (host‐induced gene silencing), plant protection, SIGS (spray‐induced gene silencing), sustainable agriculture

## Abstract

RNA interference (RNAi) has emerged as a promising tool for crop protection, offering the potential for high specificity and the ability to target a wide range of pests and pathogens through sequence‐specific design. Growing interest from academia has accelerated research in the field, while some companies have seized the opportunity to develop RNAi‐based products in regulatory environments more open to this type of innovation, positioning RNAi as a potential alternative or complement to conventional plant protection products. Notably, the recent registration of double‐stranded RNA (dsRNA)‐based products for insect control marks a transition from experimental research to early commercialization. However, several challenges remain before widespread adoption, especially in Europe. These include regulatory uncertainties, high production costs of dsRNA (used to trigger RNAi), limited comparative field efficacy data, and societal concerns related to emerging biotechnologies. Furthermore, broader validation is needed for environmental risk assessment. Despite these obstacles, RNAi has strong potential to enhance crop protection under climate change and delay resistance, especially when integrated with other technologies. Continued research, cost‐effective production methods, and strong collaborations between academia, industry and regulators are essential to support responsible implementation, potentially improving access to advanced plant protection tools for smallholders and vulnerable farming communities. © 2026 The Author(s). *Pest Management Science* published by John Wiley & Sons Ltd on behalf of Society of Chemical Industry.

## Graphical abstract

RNA interference (RNAi) technologies, host‐induced gene silencing (HIGS) and spray‐induced gene silencing (SIGS), potentially offer sustainable crop protection. However, efficacy, costs, regulatory clarity, and socio‐environmental impacts require further evaluation for broader use.

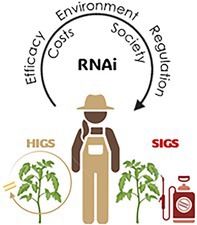

## 1 THE NEED FOR NEW PLANT PROTECTION PRODUCTS

Plant diseases pose a serious threat to food security, causing on average 21–30% yield losses on staple food crops.^1^ They can also compromise food safety by promoting the accumulation of mycotoxins and facilitating the survival of foodborne pathogens on damaged tissues (Food and Agriculture Organization of the United Nations (FAO), https://www.fao.org/one‐health/highlights/mycotoxins‐a‐silent‐risk‐to‐plants‐people‐and‐animals/en). Plant protection products (PPPs) are essential components of pest management, and without adequate crop protection, losses could reach 30–80% in major crops.^2^ Although actual losses remain significant, PPPs have enabled farmers to maintain productivity levels that would otherwise be severely compromised. However, the use of PPPs is increasingly constrained by regulatory restrictions, environmental concerns, and the progressive reduction of available active substances, particularly in the European Union (EU) under Regulation (EC) 1107/2009.^3^ As multi‐site compounds are phased out, due to non‐target toxicity, reliance on single‐site products increases the risk of resistance development in pathogen and pest populations.^4^ In this context, the need for alternatives to traditional PPPs is urgent, especially considering that many traditionally employed products, such as copper fungicides, have toxic effects and are highly persistent in the environment.^5^

Another critical issue concerns minor crops, which often include species with high nutritional value, regional economic importance, or cultural significance. Despite their relevance, the number of registered active substances available for their protection is extremely limited in Europe, leaving them particularly vulnerable to pest and disease outbreaks. This scarcity often leads to insufficient control options and increased reliance on off‐label uses or derogations (https://minoruses.eu). Considering that minor crops are worth more than 60 billion € per year, accounting for around 20% of the total EU plant production value, expanding the plant protection solutions in this sector is particularly relevant.

Moreover, beyond historically managed pathogens, the emergence of novel plant diseases driven by global trade and climate change^6^ highlights the need for effective and adaptable control tools. Recent estimates indicate that individual outbreaks, such as those caused by *Xylella fastidiosa*, *Phytophthora infestans*, and *Pyricularia oryzae*, caused losses of up to 1.2 billion USD across Europe, the Americas, and Asia in the last 5 years.^7^

In this context, the availability of innovative and targeted alternatives, such as RNA‐based solutions, can significantly broaden the toolbox for plant protection, promoting more sustainable and tailored disease management strategies.^8^

Indeed, the first RNA interference (RNAi)‐based products targeting insect pests have reached commercialization in selected markets (https://www.cropscience.bayer.us/traits/corn/smartstax-pro) and those targeting pathogens are moving toward this stage (https://greenlightbiosciences.com/articles/greenlight‐biosciences‐submits‐regulatory‐dossier‐in‐us‐eu‐and‐brazil‐for‐groundbreaking‐rna‐based‐solution‐to‐control‐grape‐powdery‐mildew). However, adoption remains uneven across regions and targets, particularly for disease control, and depends on field robustness, scalable production, regulatory alignment, and comprehensive sustainability assessment. In this context, this perspective critically examines RNAi as a crop protection tool, discussing its current position within plant protection systems and the conditions required for its effective integration into agricultural practice.

## 2 RNA INTERFERENCE FEATURES AND POTENTIAL EXPLOITATION FOR CROP PROTECTION

RNAi is a cellular mechanism that regulates gene expression through sequence‐specific silencing, mediated by small RNA molecules such as small interfering RNA (siRNA) and microRNA (miRNA). This process involves the cleavage of double‐stranded RNA (dsRNA) into siRNA, which is then incorporated into the RNA‐induced silencing complex (RISC). The siRNA guides RISC to complementary messenger RNA (mRNA) molecules, resulting in their degradation and subsequent suppression of gene expression.^9^, ^10^ In agricultural biotechnology, RNAi has emerged as a highly targeted and versatile approach to address challenges such as the management of biotic and abiotic stresses (Table 1).

**Table 1:** Non‐exhaustive list of works where the RNA interference (RNAi) has been used to control biotic and abiotic stresses

Plant species	Stressor	Gene target	Type of RNAi	Delivery method	Experimental scale	Control outcome measured	Double‐stranded RNA dose (SIGS)	Reference
*Pathogens*								
*Acer mollo*	*Verticillium dahliae*	Chitin deacetylase (*VDAG_03333*) of *V. dahliae*	SIGS	Root delivery with SPc nanocarrier via immersion	Glasshouse	Disease index	0.1 μg μL^−1^	Tao *et al*.^11^
*Brachypodium distachyon*	*Fusarium graminearum*	Protein kinase genes (*Fg00677* and *Fg08731*), cytochrome P450 lanosterol C14‐*α*‐demethylase (*CYP51*) of *F. graminearum*	HIGS	Transgenic expression	Laboratory	Quantification of the disease symptoms, *F. graminearum* biomass	Not applicable	He *et al*.^12^
*Brachypodium distachyon*	*Pyricularia oryzae*	Mitogen‐activated protein kinase (*Pmk1*) of *Magnaporthe oryzae*	SIGS	Foliar spay (with/without nanoparticles)	Growth chamber	Disease severity, relative amount of fungal DNA	1 ng μL^−1^	Zheng *et al*.^13^
*Brassica napus*	*Sclerotinia sclerotiorum*	Multiple virulence‐related genes of *S. sclerotiorum*	SIGS	Foliar spray	Laboratory	Reduced lesion size	8 ng μL^−1^	McLoughlin *et al*.^14^
*Brassica napus*	*Botrytis cinerea*	Multiple virulence‐related genes of *S. sclerotiorum*	SIGS	Foliar spray	Laboratory	Reduced lesion size	≈ 41.7 ng μL^−1^	McLoughlin *et al*.^14^
*Fragaria × ananassa*	*Botrytis cinerea*	Dicer‐like 1 (*DCL1*) and Dicer‐like 2 (*DCL2*) of *B. cinerea*	SIGS/HIGS	Spray/transgenic expression	Glasshouse	Disease severity, relative fungal biomass	50–100 ng μL^−1^ for SIGS	Capriotti *et al*.^15^
*Hordeum vulgare*	*Fusarium graminearum*	Three cytochrome P450 lanosterol C‐14*α*‐demethylases (*CYP51A*, *CYP51B*, and *CYP51C*) of *F. graminearum*	SIGS	Foliar spray	Laboratory	Lesion size, relative fungal DNA	20 ng μL^−1^	Koch *et al*.^16^
*Lactuca sativa*	*Botrytis cinerea*	MAP kinase (*BcBmp1*), tetraspanin (*BcPls1*) of *B. cinerea*	SIGS	Dropwise foliar application	Laboratory	Necrotic area	20 ng μL^−1^	Spada *et al*.^17^
*Lactuca sativa, Rosa hybrida, Solanum lycopersicum*	*Botrytis cinerea*	Vacuolar protein sorting 51 (*VPS51*), dynactin complex large subunit (*DCTN1*), suppressor of actin (*SAC1*), dicer‐like 1 and 2 (*DCL1/2*) of *B. cinerea*	SIGS	Dropwise foliar application (± LDH nanocarrier)	Laboratory	Relative lesion size	20 ng μL^−1^	Qiao *et al*.^18^
*Lactuca sativa*	*Botrytis cinerea*	MAP kinase 1 and 3 (*BcBmp1‐3*), tetraspanin (*BcPls1*) of *B. cinerea*	SIGS	Foliar spray (± nanocarrier)	Growth chamber	McKinney index, efficacy	20 ng μL^−1^	Spada *et al*.^19^
*Musa acuminata*	*Fusarium oxysporum* f. sp. *cubense* TR4	*DCL2* of *F. oxysporum*	SIGS	Root injection/injection into the rhizome area	Laboratory/glasshouse	Disease severity	2 μg for root injection, 25 ng μL^−1^ for rhizome area	Jayasekara *et al*.^20^
*Nicotiana tabacum*	*Phytophthora capsici*	RXLR effector (*PcAvr3a1*) of *Phytophthora*	HIGS	Transgenic expression	Glasshouse	Hypersensitive response	Not applicable	Vega‐Arreguín *et al*.^21^
*Oryza sativa*	*Pyricularia oryzae*	Pth11 transmembrane receptor (*MoPth11*) of *M. oryzae*	SIGS	Foliar spray (± LDH nanocarrier)	Glasshouse	Disease severity, lesion area, fungal biomass	100 ng μL^−1^	Chen *et al*.^22^
*Pinus radiata*	*Dothistroma septosporum*	Dothistromin toxin regulatory gene (*DsAflR*) of *D. septosporum*	SIGS	Foliar spray	*In vitro* culture	Lesion severity	Not specified (20 μL of dsRNA solution diluted to 1 mL)	Mosen *et al*.^23^
*Rehmannia glutinosa*	*Fusarium oxysporum*	Cytochrome P450 sterol 14*α*‐demethylase (*CYP51*), *SIX* Gene Expression 1 (*SGE1*) of *F. oxysporum*	SIGS	Foliar spray/root dipping (± nanocarrier)	Glasshouse/laboratory	Relative lesion size, fungal biomass	200 ng μL^−1^	Guo *et al*.^24^
*Solanum lycopersicum, Vitis vinifera*	*Botrytis cinerea*	Chitin (*BcCHSI*, *BcCHSIIIa*, *BcCHSVI*) and glucan (*Bcags, Bcbgs*) synthase genes of *B. cinerea*	SIGS	Fruit spray	Laboratory	Disease incidence	120 ng μL^−1^	Gebremichael *et al*.^25^
*Solanum tuberosum*	*Phytophthora infestans*	Haustorial membrane protein 1 (*PiHmp1*), cutinase (*PiCut3*) of *P. infestans*	SIGS	Foliar spray (± nanocarrier)	Laboratory, glasshouse, field	Lesion area, disease incidence, percentage of time duration protection	500 ng μL^−1^ (for laboratory), 24 mg L^−1^ for glasshouse and field	Wang *et al*.^26^
*Syzygium jambos*	*Austropuccinia psidii*	Translation elongation factor 1‐a (*EF1‐a*), and beta‐tubulin (*β‐TUB*) of *A. psidii*	SIGS	Foliar spray	Glasshouse	Disease incidence	100 ng μL^−1^	Degnan *et al*.^27^
*Vitis vinifera*	*Botrytis cinerea*	Cytochrome P450 lanosterol 14*α*‐demethylase (*BcCYP51*), chitin synthase 1 (*Bcchs1*), and Elongation factor 2 (*BcEF2*) of *B. cinerea*	SIGS	Petiole adsorption, leaf spray, post‐harvest spray	Field, laboratory	Disease severity	100 μg mL^−1^ for petiole adsorption and leaf spray, 20 μg mL^−1^ for postharvest spray	Nerva *et al* ^28^
*Vitis vinifera*	*Botrytis cinerea*	*VPS51*, *DCTN1*, *SAC1*, *DCL1/2* of *B. cinerea*	SIGS	Foliar spray (± LDH nanocarrier)	Laboratory	Relative lesion size	20 ng μL^−1^	Qiao *et al*.^18^
*Vitis vinifera*	*Plasmopara viticola*	Dicer‐like 1 and 2 (*PvDCL1/2*) of *P. viticola*	SIGS	Leaf disk dropping	Laboratory	Disease severity	75–125 ng μL^−1^	Haile *et al*.^29^
*Vitis vinifera*	*Plasmopara viticola*	*VviLBDIf7* in *V. vinifera*	SIGS (plant gene silencing)	Foliar spray	Glasshouse	Disease severity, percentage of sporulation	100 μg mL^−1^	Marcianò *et al*.^30^
*Vitis vinifera*	Esca disease	Cytochrome P450 sterol 14*α*‐demethylase family (*CYP51*) genes in *V. vinifera*	SIGS (plant gene silencing)	Trunk injection	Pots in semi‐controlled conditions	Necrotic area	10 ng μL^−1^	Nerva *et al*.^31^
*Oryza sativa*	Rice Stripe Virus (RSV)	Nucleocapsid protein (*pc3*) of RSV	SIGS	Foliar spray (± LDH nanocarrier)	Field/semi‐field	Disease incidence, symptom severity	20 μg per plant	Chen *et al*.^22^
*Phaseolus vulgaris, Nicotiana tabacum, Vigna unguiculata*	Bean Common Mosaic Virus (BCMV)	Helper component proteinase (*HC‐Pro*) and coat protein (*CP*) of BCMV	SIGS	Foliar application (± silica nanoparticles)	Growth chamber	Virus detection	230.2 μg of dsHC‐Pro and 250.5 μg of dsCP, 230.2 μg of MSP‐dsHC‐Pro	Wani *et al*.^32^
*Nicotiana tabacum*	Tobacco Mosaic Virus (TMV)	*CP* of TMV	HIGS	Transgenic expression	Glasshouse	Delay of symptom development, reduced disease incidence	Not applicable	Abel *et al*.^33^
*Cucurbita pepo*	Tomato leaf curl New Delhi virus (ToLCNDV)	All the open reading frames (ORFs) present in the DNA‐A of ToLCNDV	SIGS	Dropwise foliar application	Growth chamber	Virus detection	100 μg	Frascati *et al*.^34^
*Vitis vinifera*	Grapevine Pinot Gris Virus (GPGV)	RNA‐dependent RNA polymerase (*RdRp*) in GPGV	SIGS	Shoot tip dipping	*In vitro* (tissue culture)	Virus detection	1200 ng μL^−1^	Kaur *et al*.^35^
*Zea mays*	Sugarcane Mosaic Virus (SCMV)	*CP* gene of SCMV	SIGS	Foliar spray (bacterial extracts)	Glass house	Disease incidence	~3 μg μL^−1^ (crude extract)	Gan *et al*.^36^
*Pests*								
*Glycine max*	*Aphis glycines*	Soluble trehalase (*TREH*), V‐type proton ATPase subunit D (*ATPD*), V‐type proton ATPase subunit E (*ATPE*), chitin synthase 1 (*CHS1*) of *A. glycines*	SIGS	Foliar spray/dropwise root application with nanocarrier	Laboratory	Percent mortality	5000 ng μL^−1^ for foliar spray, 500 μg dsRNA per plant for root application	Yan *et al*.^37^
*Phaseolus vulgaris*	*Apolygus lucorum*	Ecdysone receptor (*ECR‐A*), trehalase (*Tre‐1*) of *Apolygus lucorum*	SIGS	Topical application on insects/spray application on plants with nanocarriers	Controlled environment	Pest mortality	250 ng μL^−1^	Qiao *et al*.^38^
*Solanum tuberosum*	*Leptinotarsa decemlineata*	Actin of *L. decemlineata*	SIGS	Leaf disk assay	Laboratory	Pest mortality	900 ng μL^−1^	Darrington *et al*.^39^
*Solanum tuberosum*	*Leptinotarsa decemlineata*	Proteasome Subunit Beta Type‐5 (*PSMB5*) of *L. decemlineata*	SIGS	Leaf immersion	Glasshouse	Percent defoliation	03–4.9 g ha^−1^	Rodrigues *et al*.^40^
*Solanum tuberosum*	*Leptinotarsa decemlineata*	*PSMB5* of *L. decemlineata*	SIGS	Leaf immersion	Laboratory	Mortality	0.2 g L^−1^	Graser *et al*.^41^
*Solanum tuberosum*	*Leptinotarsa decemlineata*	Mesh (dsMESH) of *L. decemlineata*	SIGS	Foliar spray	Field trial	Larvae count, stage determination, leaf damage	10 μg mL^−1^	Petek *et al*.^42^
*Zea mays*	*Diabrotica virgifera virgifera*	Vacuolar ATPase subunit A (*V‐ATPase A*) of *D. virgifera*	HIGS	Transgenic expression	Growth chamber	Reduced feeding damage, larval stunting, mortality	Not applicable	Baum *et al*.^43^
*Abiotic stress*								
*Triticum aestivum*	Drought	Proline dehydrogenase gene (*pdh*) of *T. aestivum*	HISG	Transgenic expression	Pots	Biochemical responses	Not applicable	Dubrovna *et al*.^44^
*Vitis vinifera*	Drought	Glutathione S‐transferase (*VvGST40*) of *V. vinifera*	SIGS	Foliar spray	Pots in open air conditions	Ecophysiological, biochemical and molecular responses	50 μg	Nerva *et al*.^45^
*Postharvest*								
*Manihot esculenta*	Postharvest physiological (PPD) deterioration	Feruloyl CoA 6′‐hydroxylase genes (*F6′H*) of *Manihot esculenta*	HIGS	Transgenic expression	Glasshouse	Reduced scopoletin accumulation, delayed PPD discoloration	Not applicable	Liu *et al*.^46^
*Solanum tuberosum*	Browning of fresh‐cut	Polyphenol oxidase (*StPPO*), phenylalanine ammonia‐lyase (*StPAL2*) of *S. tuberosum*	SIGS	Postharvest spray on tubers	Laboratory	Brown index	0.1 g L^−1^	Chen *et al*.^47^

*Note*: Works in which RNAi or spray‐induced gene silencing (SIGS) was applied exclusively to isolated pathogens or pests, without experimental evidence of interaction with the plant host, were not considered. HIGS, host‐induced gene silencing. Species names are updated to current taxonomy; names in original studies may differ.

**Figure 1:**
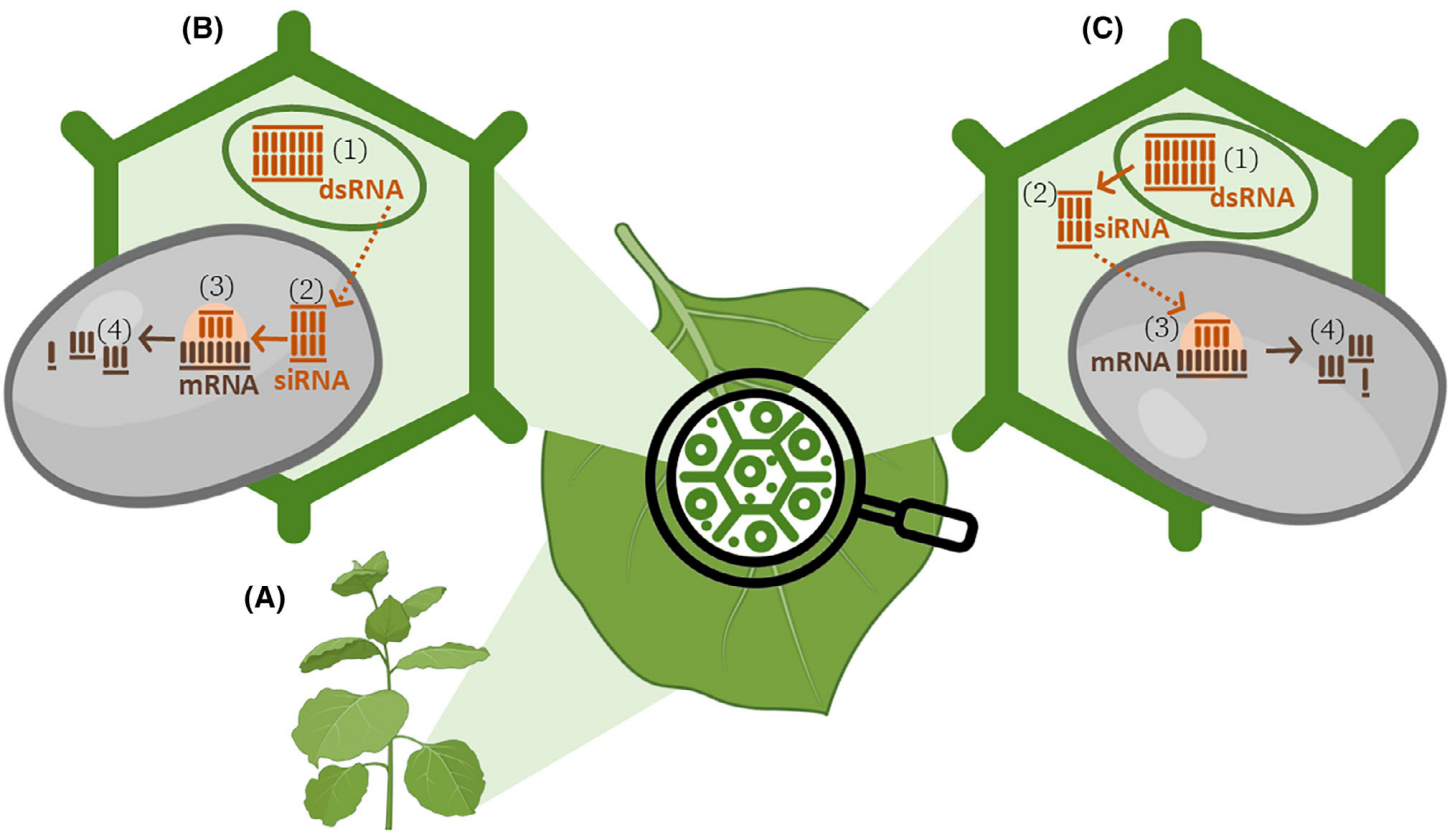
(A) A genetically engineered plant constitutively produces double‐stranded RNA (dsRNA) targeting pathogen genes, eliminating the need for external RNA applications. The mechanisms underlying the transfer of silencing RNAs across the plant–pathogen interface remain incompletely resolved (dashed arrows) and may involve the uptake of either dsRNA or processed small interfering RNAs (siRNAs), depending on the pathosystem (e.g., Koch *et al*.^16^ and Wang *et al*.^48^). (B) On the left, dsRNA synthesized in the plant nucleus (1) is transferred to the pathogen and processed into siRNAs (2) by the pathogen RNAi machinery. These siRNAs bind complementary target messenger RNAs (mRNAs) (3) and direct their cleavage (4). (C) On the right, dsRNA produced in the plant cell nucleus (1) is processed into siRNAs (2) within the plant cell, which are then taken up by the pathogen and bind to target mRNAs (3), leading to their cleavage (4). In both cases, degradation of pathogen mRNAs results in the silencing of genes involved in infection, thereby reducing disease development. The figure was created by modifying a template from BioRender.com.

RNAi technologies can be deployed through two primary strategies: transformative and non‐transformative gene silencing. The transformative strategy, also referred to as host‐induced gene silencing (HIGS) (Fig. 1),^16^, ^48^ relies on the stable genetic modification of plants to produce dsRNA specifically designed to target and silence specific genes.

**Figure 2:**
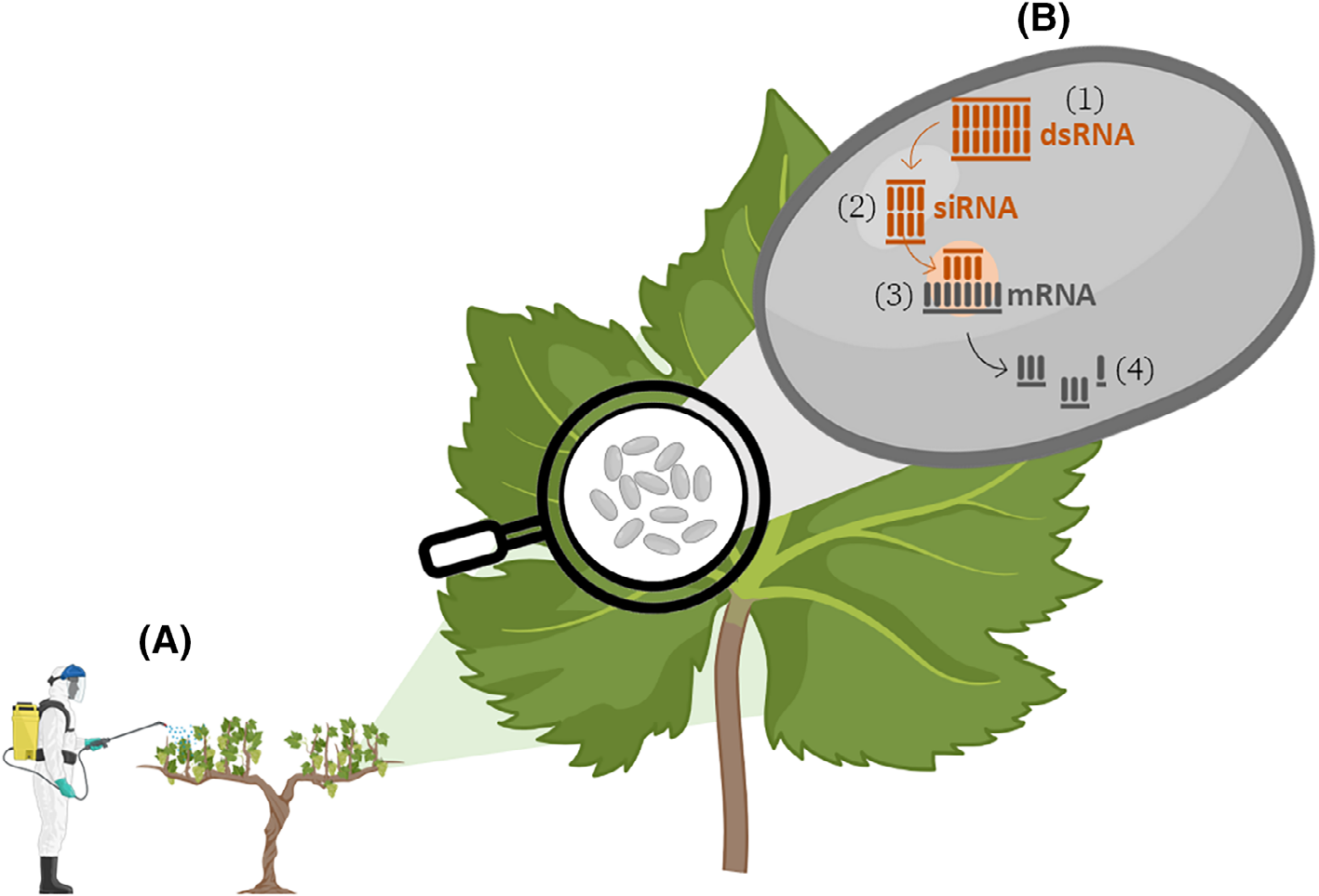
Overview of spray‐induced gene silencing (SIGS) strategies for disease control. (A) Double‐stranded RNA (dsRNA) is applied to the plant surface as a foliar spray. (B) On the right, a pathogen structure (gray) has taken up the dsRNA (1), which is processed into small interfering RNAs (siRNAs) (2). The siRNAs bind to their complementary target messenger RNA (mRNA) (3) and direct its cleavage (4), leading to the silencing of pathogen genes involved in infection. Degradation of pathogen target mRNAs reduces disease development by impairing pathogen growth. The figure was created by modifying a template from BioRender.com.

In contrast, the non‐transformative strategy, known as spray‐induced gene silencing (SIGS), involves the external application of dsRNA, enabling uptake by plants without genetic modification (Fig. 2).^49^, ^50^ Both strategies have been successfully applied, at least in controlled conditions, to mitigate major plant stress factors, mostly by targeting pathogen and pest essential genes, confirming the versatility of RNAi‐based approaches in disease and pest management (Table 1).^51^ The rapidly growing body of literature (634 publications retrieved from Scopus using the keywords ‘RNA interference’ AND ‘plant protection’, 330 of which were published between 2020 and 2025 – accessed on 16 February 2026) reflects the strong academic interest and extensive investigation into the potential applications of RNAi in plant protection. Notably, 178 of these publications are review articles, highlighting the intense effort devoted to synthesizing and consolidating knowledge in this rapidly evolving field. This sustained scientific attention is largely driven by the intrinsic advantages of RNAi‐based approaches. Unlike conventional products, which often have broad‐spectrum effects and can harm non‐target organisms, RNAi silences specific genes, minimizing ecological harm and enhancing environmental compatibility.^51^ Indeed, dsRNA targeting essential insect genes have been shown to reduce pest populations without affecting beneficial non‐target species such as pollinators.^52^, ^53^ At the same time, recent regulatory approvals and industrial investments demonstrate that the technology is progressively entering an early commercialization phase. In this context, a forward‐looking perspective examining how RNAi technologies can be realistically integrated into crop protection systems, including resistance management and regulatory constraints, becomes relevant. Beyond pest management, RNAi serves as a powerful tool for crop improvement by enabling the precise targeting of specific plant genes to enhance desirable traits, such as drought tolerance^45^ and greater nutrient use efficiency.^54^ These applications are particularly valuable in the context of climate change and the need for sustainable food production.^49^, ^50^

Table 1 provides a representative, non‐exhaustive overview of RNAi‐based strategies in which dsRNA application was evaluated in the context of plant–stressor interactions (pathogens and pests for biotic stresses, drought for abiotic stress and postharvest physiological deterioration). The studies span different biological targets, delivery strategies, and experimental scales, ranging from laboratory and controlled‐environment assays to semi‐field or field studies. Importantly, the evidence summarized in Table 1 reveals substantial variability in the dsRNA doses required to achieve measurable biological effects, with reported amounts spanning several orders of magnitude and expressed using different metrics (e.g., ng μL^−1^, μg per plant, g ha^−1^). Despite the growing body of literature, most RNAi applications remain at the proof‐of‐concept stage, with consistent field‐level management documented only in specific cases (see Section 5).^28^ While commercialization has been achieved for selected arthropod targets, applications against plant pathogens remain largely emerging and require further validation for broad deployment.

Depending on the nature of the stressor, RNAi target genes span a broad functional spectrum, including pathogen genes essential for growth, virulence, or infection establishment,^13^, ^14^ genes encoding viral structural components,^22^ insect genes involved in metabolism or development,^37^, ^38^ or stress‐related genes.^44^, ^47^

**Figure 3:**
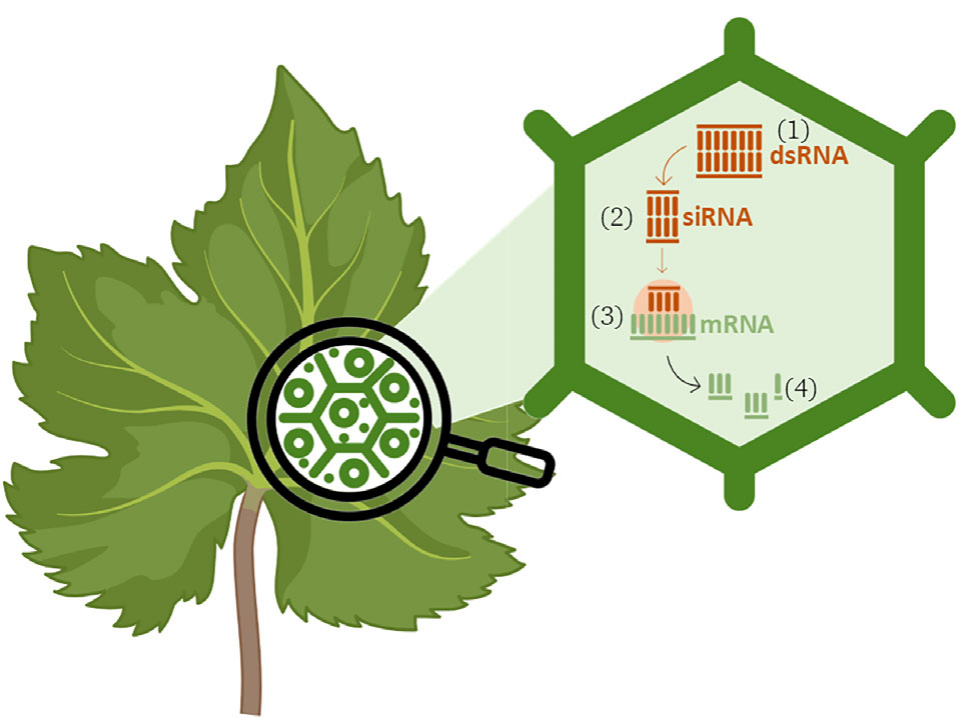
RNA interference (RNAi)‐mediated silencing of plant susceptibility (S) genes for disease resistance. Double‐stranded RNA (dsRNA), either produced endogenously in the plant or applied exogenously, targets plant S genes involved in pathogen infection. Following production or uptake, dsRNA (1) is processed by the plant RNAi machinery into small interfering RNAs (siRNAs) (2). These siRNAs bind complementary plant target messenger RNAs (mRNAs) (3) and guide their cleavage or translational repression (4). Silencing of host susceptibility genes reduces compatibility between plant and pathogen, thereby enhancing disease resistance. The figure was created by modifying a template from BioRender.com.

An additional and still underexploited opportunity lies in the use of dsRNA to target plant susceptibility (S) genes,^30^, ^31^ rather than genes of the pathogen (Fig. 3). S‐genes encode host factors that are exploited by pathogens to facilitate infection, colonization, or disease progression. Although S‐gene silencing has been explored using genetic approaches, such as genome editing, its implementation via SIGS remains limited. From a conceptual standpoint, targeting S‐genes offers several advantages: it may confer reduced host susceptibility, potentially improving the durability of resistance. The dsRNA‐based targeting of S‐genes could represent a complementary or alternative strategy to direct pathogen silencing, especially in complex pathosystems or where pathogen uptake of dsRNA is inefficient. When integrated with approaches targeting pathogen or pest genes, S‐gene silencing could contribute to multi‐layered protection strategies, enhancing robustness while maintaining the specificity and environmental compatibility of RNAi‐based crop protection.

Since SIGS is currently the most investigated and practically applicable RNAi strategy for agricultural use, the following sections will primarily focus on this approach, while HIGS will be discussed where relevant.

## 3 KEY FACTORS DETERMINING THE EFFICACY OF SIGS‐BASED RNA INTERFERENCE APPROACHES

The efficacy of RNAi depends on multiple factors, including the design of the dsRNA, host plant characteristics, target organism biology, and the delivery method.^55^ In the context of SIGS, these factors are particularly critical, as exogenously applied dsRNA must remain stable in the environment, be taken up efficiently, and reach its target.

### 3.1 Target selection and dsRNA design

Among the various factors, dsRNA design is critical to ensure high specificity and effectiveness of the treatment. The dsRNA can be designed to target specific genes, required for growth, development, or virulence in biotic stress agents, or alternatively plant endogenous genes involved in susceptibility to disease. In both cases, selecting highly specific gene sequences helps minimize off‐target effects on non‐target organisms or unintended plant genes. Several bioinformatic tools, such as dsRNAEngineer and SiFi, have been developed to predict potential off‐target events and optimize sequence selection.^56^, ^57^ It is also possible to use single‐gene or multi‐gene targeting approaches. These choices are critical for optimizing both specificity and long‐term effectiveness, especially in reducing the likelihood of resistance development in the target organism.^58^ Another critical parameter is dsRNA length, which depends on the target species and the delivery method. Although uptake mechanisms remain incompletely understood,^59^ efficient processing by Dicer enzymes appears to require dsRNA molecules within a defined length range, and dsRNAs of 200–1000 base pairs are generally recommended to ensure efficient silencing.^55^

### 3.2 Environmental stability and formulation strategies

After foliar application, dsRNA must remain sufficiently stable on the plant surface to contact the target organism. Environmental factors such as ultraviolet (UV) radiation, precipitation, and microbial activity can reduce stability under field conditions, limiting RNAi persistence. While rapid degradation is environmentally advantageous, it may compromise efficacy if uptake does not occur. To address these limitations, delivery strategies have been developed to enhance stability and bioavailability. Encapsulation approaches are a major research focus for improving RNAi performance under field‐relevant conditions. Carriers including chitosan, liposomes, and clay nanosheets can protect dsRNA from degradation and improve uptake^60^, ^61^, ^62^ Several studies summarized in Table 1 report enhanced and prolonged activity when dsRNA is combined with carrier materials or nanoparticle‐based formulations compared with naked dsRNA applications.^13^, ^18^, ^32^ Biological systems such as yeast or *Escherichia coli*, have also been explored for dsRNA production and delivery.^63^ These approaches aim to balance environmental compatibility with sufficient persistence for effective silencing.^64^

### 3.3 Target‐specific uptake constraints

The success of SIGS depends on the ability of dsRNA to be internalized within the biological compartment where silencing occurs. Uptake requirements differ depending on whether the target is a pest or pathogen gene, or a plant susceptibility gene.

#### 3.3.1 Targeting pest or pathogen genes

When targeting pest or pathogen genes, efficacy depends on uptake by the target organism and activation of its RNAi machinery. In insects, silencing requires ingestion or contact‐mediated acquisition, stability within the gut environment, translocation across epithelial barriers, and intracellular processing.^65^, ^66^, ^67^ Responsiveness varies markedly across taxa: Coleoptera are often considered among the most responsive groups, although susceptibility is species‐dependent; Hemiptera show variable responses; and Lepidoptera frequently display lower responsiveness to exogenous dsRNA.^68^ In fungi, efficient uptake and disease reduction have been demonstrated in species such as *Botrytis cinerea* ^28^ and uptake has also been reported in *Sclerotinia sclerotiorum*, *Rhizoctonia solani*, *Aspergillus niger*, and *Verticillium dahliae*.^3^ In contrast, limited or no uptake has been observed in other taxa, including certain *Colletotrichum* species, and oomycetes generally exhibit lower levels of dsRNA internalization.^69^ These observations indicate that SIGS efficacy cannot be generalized across biological groups, but must be evaluated within each specific pest or pathosystem.

#### 3.3.2 Targeting plant susceptibility genes

S‐gene targeting primarily depends on delivery into plant cells and activation of the endogenous RNAi machinery. Following foliar application, dsRNA must cross the cuticle and epidermal layers, overcome the cell wall and plasma membrane, and avoid degradation by plant nucleases.^70^ These structural and biochemical barriers are influenced by plant physiology, developmental stage, and species‐specific traits.^71^ As a consequence, different plant species, and even different cultivars, may vary in their responsiveness to SIGS‐based strategies, making tissue accessibility a central constraint in this context. This variability should be taken into account when designing RNAi‐based treatments targeting plant S‐genes. In addition to cellular entry, systemic movement of dsRNA or derived siRNAs through the vascular system may significantly influence the overall effectiveness of the treatment.^70^ Translocation from the site of application to distal tissues could enhance coverage and durability of silencing, particularly in perennial crops or when infection occurs in tissues not directly exposed to spraying.^72^

## 4 ENVIRONMENTAL, ECONOMIC, AND SOCIAL SUSTAINABILITY OF RNA INTERFERENCE

### 4.1 Impact of dsRNA on the environment

While PPPs are effective tools for increasing agricultural productivity, their use is limited by a combination of environmental impacts, health risks, economic challenges, regulatory frameworks, and societal preferences, which are driving the shift toward more sustainable and innovative agricultural practices. Specifically, the use of PPPs involves an environmental impact mainly due to the mechanization of PPPs applications, the consumption of PPPs (whose manufacturing is energy‐intensive), and the emissions of active ingredients and co‐formulants into air, water, and soil.^73^

As previously highlighted, RNAi‐based strategies represent a promising sustainable and eco‐friendly solution to address the challenges of modern agriculture. Unlike conventional chemical PPPs, dsRNA‐based approaches rely on a highly specific and biodegradable mechanism, offering several environmental advantages. Conventional chemical PPPs often persist in the environment, leach into soil and water systems, and cause long‐term contamination.^74^, ^75^ In contrast, dsRNA molecules are naturally degraded by nucleases present in the environment. This rapid biodegradation minimizes the risk of accumulation in ecosystems, reducing the potential for soil and water pollution. Studies have shown that naked dsRNA degrades rapidly in soil, aquatic systems and plants, minimizing potential environmental accumulation.^76^ Indeed, dsRNA exhibited half‐life (DT_50_) values in soils ranging from 15 to 28 h and DT_90_ (time to 90% degradation) values of less than 35 h. These data indicate that dsRNAs undergo rapid degradation, with their biological activity becoming undetectable within approximately 2 days after application, regardless of soil characteristics such as texture, pH, or clay content.^77^ Similarly, the degradation dynamics of dsRNA in aquatic systems appear to mirror those observed in soils, with an estimated DT_50_ of less than 3 days and a DT_90_ of approximately 4 days.^78^, ^79^ The environmental fate and persistence of foliarly‐applied dsRNA remain poorly understood, with existing studies presenting inconsistent results.^76^ Under controlled conditions, Mitter *et al*.^80^ reported that dsRNA sprayed on leaf surfaces provided only 5 days of virus protection before degrading. In contrast, San Miguel and Scott^81^ observed insecticidal dsRNA efficacy on potato leaves for up to 28 days. Contrasting results have also been reported regarding the impact of rainfall on the wash‐off of foliarly‐applied dsRNA. According to Mitter *et al*.,^80^ dsRNAs rapidly washed off after a simulated rainfall 24 h post‐application. In contrast, San Miguel and Scott^81^ demonstrated that dsRNA remained active for up to 28 days, likely due to its adherence to the leaf surface after drying. In field conditions, foliarly‐applied dsRNA declined by approximately 95% within 3 days and approximately 99% within 7 days after application, with dissipation kinetics estimating a DT_50_ of 0.7–0.5 days and a DT_90_ of 2.3–1.9 days.^76^

The use of stabilized formulations and nanocarrier‐based delivery systems,^18^, ^61^ previously discussed as strategies to enhance dsRNA performance, may also influence the dsRNA environmental fate. By increasing protection against UV radiation, wash‐off, and microbial degradation, these formulations can extend molecular persistence in soil and on plant surfaces. For instance, soil stability tests have shown that linear cationic polymers can delay dsRNA degradation for up to 1 week, while star‐shaped polymeric structures may prolong persistence for up to 3 weeks.^82^ Despite the enhanced stability in soil, the persistence of formulated dsRNA remains consistently lower than that of conventional agrochemicals, although further studies are essential to fully understand its environmental dynamics and ensure the safe deployment of these innovative agricultural tools.

Another challenge in these highly specific applications is minimizing off‐target effects on non‐target organisms.^17^, ^31^ Currently, few studies have analyzed the effects of specific treatments using RNAi on non‐target organisms, particularly overlooking the potential impacts on beneficial species. Future research should also address these aspects to enable the application of such treatments with a comprehensive understanding of their effects on the entire ecosystem surrounding the plant.

### 4.2 Cost‐effectiveness and yield optimization in dsRNA production

The large‐scale application of RNAi in agriculture depends largely on the ability to produce high‐quality dsRNA in sufficient quantities at affordable costs. Achieving production costs within a range compatible with those of conventional chemical PPPs, which often range from 10 to 100 € per gram of active ingredient,^83^ is an important prerequisite for the market competitiveness of dsRNA‐based solutions. Several authors have indicated in review articles that chemical synthesis, although accurate, is costly and inefficient for the production of long dsRNA molecules (Table 2), generates hazardous waste and requires complex purification steps.

**Table 2:** Comparison of costs (USD) and yield of different double‐stranded RNA (dsRNA) production methods

Production system	Estimated cost per gram (USD)	Yield	Notes	Reference
Chemical synthesis	3000–10000^†^	1 g	Precise, but costly and polluting	da Rosa *et al*.^84^
Cell‐free enzymatic synthesis (industrial proprietary platform)	< 1 (reported by company sources)	Industrial scale (hundreds of kg yr^−1^ year; g L^−1^ not disclosed)	Proprietary large‐scale enzymatic production platform; data based on publicly available company information	https://greenlightbiosciences.com/how‐do‐we‐make‐rna
*Escherichia coli* (optimized)	< 250	0.05–0.2 g L^−1^	Scalable and cost‐efficient	Guan *et al*.^85^ and da Rosa *et al*.^84^
*Corynebacterium glutamicum*	1–4	< 1 g L^−1^	Scalable and cost‐efficient	Hashiro *et al*.^86^
*Yarrowia lipolytica*	Unknown	0.2 μg L^−1^	Can use alternative carbon sources	Álvarez‐Sánchez *et al*.^87^

^†^ In case of long dsRNA sequences (> 1500 bases).

Recent industrial developments indicate that the production cost of dsRNAs can be markedly reduced. For example, the dsRNA active ingredient ledprona, developed by GreenLight Biosciences for the Colorado potato beetle control, has been reported to reach projected production costs below 1 USD per unit of dsRNA at the synthesis stage. The patent owned by GreenLight protects the production process of the active ingredient; however, the 490 bp dsRNA is produced using the proprietary cell‐free bioprocessing platform.^40^, ^88^

Biotechnological synthesis of dsRNA offers a scalable and cost‐effective alternative to chemical synthesis. Microorganisms such as *E. coli* and *Bacillus subtilis*, which can be genetically engineered to maximize dsRNA production, have been explored for dsRNA production through both *in vivo* expression and *in vitro* transcription.^85^, ^89^ These systems enable substantial cost reductions, with projected production costs falling below 1 USD per gram and, in optimized cell‐free systems, potentially reaching approximately 0.50 USD per gram (Table 2).^90^ The success of biotechnological approaches, however, depends on the optimization of culture media and induction strategies.^84^, ^90^

The use of glycerol‐based media enhances bacterial growth and, similarly, replacing conventional LB medium with Terrific Broth (TB) can increase dsRNA production by 118%, while lactose induction (6 g L^−1^) has been shown to yield up to ten‐fold higher dsRNA levels than isopropyl *β*‐d‐1‐thiogalactopyranoside (IPTG) induction.^90^ Furthermore, optimization of growth conditions and fermentation parameters is critical for maximizing production, and similar gains resulting from fermentation optimization have been reported in other microbial systems, including *Perkinsus marinus*.^91^

Yeast‐based systems, particularly *Saccharomyces cerevisiae* offer several advantages for large‐scale dsRNA production since they can be easily genetically manipulated and are capable of producing highly pure dsRNA without the need for extensive downstream processing. Moreover, large‐scale fermentation plants are already available. Genetically engineered *S. cerevisiae* strains yield over 1.2 g L^−1^ of dsRNA when cultivated under optimized conditions, such as YPD (yeast extract, peptone, dextrose) medium supplemented with galactose, temperature controlled at 30 °C and pH stabilized at 6.5.^63^ *Yarrowia lipolytica*, can utilize a broader range of carbon sources, including hydrophobic substrates, has also been explored for dsRNA production: engineered strains of *Y. lipolytica* can produce dsRNA yields comparable to *S. cerevisiae* (Table 2), using nitrogen‐limited conditions and oleic acid induction.^87^

Plants represent promising biofactories for the production of dsRNA, offering several advantages including scalability, cost‐effectiveness, and the ability to generate substantial amounts of RNA directly *in planta* through transient or stable expression systems.^92^ Although transgene‐derived dsRNA can be detected and quantified *in planta*, plants are not yet routinely exploited for the large‐scale production and purification of long, intact *in vivo*‐derived dsRNA molecules.^93^ One major limitation lies in the endogenous RNA interference machinery, particularly Dicer‐like enzymes, which process long dsRNA into siRNAs, thereby reducing the accumulation of unprocessed dsRNA.^94^ Nevertheless, recent advances in molecular engineering and RNA stabilization strategies suggest that novel approaches may enable the accumulation and recovery of longer, minimally processed dsRNA molecules *in planta*, opening new perspectives for plant‐based dsRNA production systems.

Ultimately, the selection of production systems should be guided by a balanced evaluation of economic viability and environmental sustainability to ensure responsible large‐scale implementation.

### 4.3 Social sustainability

Social sustainability is about the satisfaction of basic human needs and the provision of the rights and the freedoms to satisfy one's aspirations for a better life.^95^ Within the Framework for Strategic Sustainable Development (FSSD), the social dimension has long been the least explored.^96^ In the context of agriculture, innovations such as RNAi‐based technologies could potentially contribute to different aspects of social well‐being themes identified by FAO within the Sustainability Assessment of Food and Agriculture (SAFA) systems guidelines (Table 3).^97^ The themes include decent livelihood, fair trading practices, equity, human safety and health, and food sovereignty. The possibility of exploiting HIGS and SIGS at relatively low cost may facilitate access to effective disease control tools even for smallholders or farmers cultivating minor crops, thereby improving access to the means of production and supporting livelihood security (S1 – Decent Livelihood: fair access to means of production). Furthermore, if RNAi technologies are developed within public research frameworks or released without restrictive patents, they could reduce the risk of exploitative dependencies along the supply chain, supporting fairer trading conditions and strengthening the position of suppliers (S2 – Fair Trading Practices: rights of suppliers). The adoption of RNAi may also benefit socially vulnerable groups by reducing the physical workload and exposure to harmful chemicals traditionally associated with PPP application, especially when using HIGS (S4 – Equity: support to vulnerable people). These improvements are particularly relevant in contexts where women, elderly farmers, or laborers from disadvantaged communities play a role in agricultural production. From a health perspective, the reduced use of conventional fungicides may lower the risk of acute and chronic intoxication among workers (S5 – Human Safety and Health: workplace safety and health) and limit PPP residues in food and the environment, contributing to improved human and environmental health outcomes (S5 – Human Safety and Health: public health). A growing societal concern that increasingly shapes consumer preferences and agricultural policies, particularly in Europe, is the aversion to conventional chemical PPPs, commonly referred to as chemophobia.^98^ While this fear is not always scientifically justified, it highlights a broader public demand for transparency and safer alternatives in crop protection.^99^ In this context, RNAi‐based solutions could help address such concerns by offering highly specific, non‐toxic, and biodegradable tools for disease management. It is important, however, to distinguish between SIGS and HIGS. While SIGS relies on exogenous application of dsRNA and does not involve permanent genetic modification of the plant, HIGS is based on stable genetic transformation and therefore falls within existing genetically modified organism (GMO) regulatory frameworks. Consequently, the public acceptance of RNAi technologies may differ substantially depending on the implementation strategy. The perceived ‘naturalness’ of dsRNA, which does not involve permanent genetic modification nor toxic residues, may facilitate public acceptance. Public perception of HIGS‐based approaches is likely to reflect broader societal attitudes toward genetically modified crops, which have been reported as cautious or divided in several European countries.^3^, ^98^

**Table 3:** Social well‐being aspects identified by the Food and Agriculture Organization of the United Nations (FAO) Sustainability Assessment of Food and Agriculture (SAFA) systems that could be potentially satisfied by RNA interference (RNAi) technologies in plant disease management

Theme	Aspect	Aspect satisfied by RNAi technologies
S1 – Decent Livelihood	Fair access to means of production	Potential to develop tailored RNAi solutions for a wide range of crops and diseases can improve economic viability for farmers
S2 – Fair Trading Practices	Rights of suppliers	If RNAi technologies are not patent‐restricted or are developed within public frameworks, they may avoid exploitative dependencies and promote fairer value chains
S4 – Equity	Support to vulnerable people	By reducing workload and input costs, RNAi (HIGS) can support smallholders, women, and elderly farmers, contributing to more equitable farming systems
S5 – Human Safety and Health	Workplace safety and health	RNAi‐based crop protection (HIGS and SIGS) reduces farmers' exposure to hazardous PPPs, improving occupational health and safety
	Public health	Reduced PPP use leads to lower environmental contamination and fewer residues in food, supporting better public health outcomes
S6 – Food Sovereignty	Access to technology and knowledge	RNAi technologies (HIGS) can increase local control over crop protection, reducing dependency on external inputs, and enhancing food sovereignty, particularly in developing countries

*Note*: HIGS, host‐induced gene silencing; PPP, plant protection product; SIGS, spray‐induced gene silencing.

Finally, broader access to RNAi technologies could enhance farmers' autonomy in managing plant diseases, especially in low‐ and middle‐income countries. This would reduce reliance on external inputs and align with the principles of food sovereignty, by increasing disease control tools and crop protection strategies (S6 – Cultural diversity: food sovereignty). Indeed, the relevance of RNAi‐based technologies in supporting smallholder farmers has been recently emphasized in a dedicated perspective, which highlighted their potential to contribute to the sustainable intensification of agriculture in resource‐limited contexts, where the simultaneous need to reduce input demands and environmental impacts while complementing traditional practices must be addressed.^100^

## 5 CURRENT STATUS ON REGISTRATION AND USE

Due to their potentially high specificity and favorable safety profile, RNAi‐based products could face fewer regulatory hurdles compared to conventional chemical pesticides, potentially facilitating their registration.^8^

However, the regulatory landscape varies significantly across regions, with notable differences in the approval processes for HIGS and SIGS applications. While the United States has approved RNAi‐utilizing products for agricultural applications, exemplified by the 2017 approval of a genetically modified maize event (MON87411) utilizing HIGS technology, this has not been uniformly adopted worldwide. Notably, both in the United States and Europe, these products fall under the purview of GMO regulations. However, the EU has approved this maize event only for non‐agricultural uses.^101^ This product integrates two *Bacillus thuringiensis* (Bt) proteins with the *DvSnf7* cassette, which produces dsRNAs specifically targeting the *Snf7* genes of the western corn rootworm (*Diabrotica virgifera virgifera*).^102^

In Europe, HIGS plants fall under the directive of (EC) 2001/18 or (EC) 1829/2003, depending on whether they are intended for any use or specific food and feed use respectively. In August 2024, the European Food Safety Authority (EFSA) approved maize MON95275 for full‐scale use in the food and feed industries. This genetically modified plant is distinct from MON87411 due to the insertion of the *DvSnf7* cassette.^103^ In January 2025, EFSA completed a 3‐year project aimed at improving risk assessment methodologies for RNAi‐based genetically modified plants.^104^ The resulting opinion confirmed that existing frameworks are generally adequate for HIGS plants. While ingestion of dsRNAs through diet is considered unlikely to pose health risks, environmental risk assessments should account for sequence‐specific interactions and the limitations in predicting RNAi activity across diverse species.

A different regulatory scenario applies to SIGS, due to its exogenous application. In the United States, dsRNA products are classified as biochemical pesticides^8^ and regulated by the Environmental Protection Agency (EPA), under the Federal Insecticide, Fungicide, and Rodenticide Act (FIFRA) and the Federal Food, Drug, and Cosmetic Act (FFDCA). In 2023, the EPA approved the first dsRNA‐based insecticide, ledprona, targeting the Colorado potato beetle. The product is registered as a biopesticide (EPA Reg. No. 94614‐2) and represents a major milestone in RNAi commercialization. A dsRNA‐based product targeting the Varroa mite (vadescana) has also reached commercialization. More recently, a dossier has been submitted in the United States, EU, and Brazil for a dsRNA active ingredient targeting *Erysiphe necator* (https://greenlightbiosciences.com/articles/greenlight‐biosciences‐submits‐regulatory‐dossier‐in‐us‐eu‐and‐brazil‐for‐groundbreaking‐rna‐based‐solution‐to‐control‐grape‐powdery‐mildew), marking one of the first regulatory submissions for fungal disease management. This indicates that RNAi applications for fungal disease management are progressing beyond proof‐of‐concept studies toward formal regulatory evaluation. While authorization has not yet been granted, such submissions mark an important step in assessing the technological readiness of SIGS‐based solutions for pathogen control.

In Europe, SIGS products are regulated differently depending on their composition. If they contain living GMOs, they fall under the purview of the GMOs Regulation (EC) 1829/2003,^101^ whereas non‐GMO dsRNA products are assessed under Regulation (EC) 1107/2009. The authorization process follows a two‐stage pathway: EU‐level approval of the active substance by EFSA and the European Commission, followed by zonal evaluation of the PPP by Member States.^105^ Although dsRNAs may be considered a new class of active substances under Regulation (EC) 283/2013, in practice they are currently assessed within the framework developed for chemical PPPs.

The regulatory landscape in other regions follows similar distinctions. HIGS products are treated as GMOs and evaluated by national biosafety authorities, including the Comissão Técnica Nacional de Biossegurança (CTNBio) in Brazil, the Environmental Protection Authority (EPA) based on the Hazardous Substances and New Organisms Act 1996 (HSNO Act) in New Zealand, the Office of the Gene Technology Regulator (OGTR) (Gene Technology Act 2000) in Australia and the Genetic Engineering Appraisal Committee (GEAC) (Rules for the Manufacture, Use/Import/Export and Storage of Hazardous Micro Organisms/Genetically Engineered Organisms or Cells, 1989) in India. In contrast, SIGS generally benefits from a simpler regulatory pathway and broader public acceptance in many countries. In Brazil, for example, it may be subject to regulation as a new biopesticide/PPP, separately from GMOs, but regulatory specificity is evolving (Lei de Biossegurança n. 11.105/2005). In Australia, it is under discussion: it is unlikely to be classified as a GMO by the OGTR, but would likely be regulated as an agrochemical/biopesticide by the APVMA (Australian Pesticides and Veterinary Medicines Authority, Agricultural and Veterinary Chemicals (Administration) Act 1992).

Globally, a distinction is emerging between transgenic HIGS approaches, subject to GMO regulation, and spray‐applied SIGS strategies, which may follow pesticide regulatory pathways. While this differentiation may facilitate authorization and public acceptance of SIGS, it does not guarantee rapid market uptake, particularly in the EU, where dsRNA‐based products are still assessed within regulatory frameworks originally designed for chemical active substances and where long‐term environmental and societal acceptance remain critical determinants.

## 6 EMERGING AND UNDEREXPLORED OPPORTUNITIES FOR NEXT‐GENERATION DOUBLE‐STRANDED RNA‐BASED CROP PROTECTION

Despite the substantial progress achieved by dsRNA‐based technologies, their application still presents limitations that can be reframed as opportunities for innovation. Next, we outline several emerging and underexplored directions that could expand the functional scope and practical relevance of dsRNA‐based crop protection beyond current single‐target implementations.

### 6.1 Multi‐target and multi‐disease protection through dsRNA complexes

The dsRNA current implementation largely relies on a single‐target, single‐stressor paradigm, in which individual dsRNA molecules are designed to silence one gene in one pathogen or pest. While this approach has been essential to demonstrate feasibility, it may limit the broader potential of RNAi in complex agricultural systems, where multiple stresses often co‐occur. One of the most promising yet largely unexplored opportunities is the development of dsRNA complexes or cocktails capable of targeting multiple genes and/or multiple stressors simultaneously. Designed dsRNA complexes could enable the simultaneous targeting and silencing of multiple pathogens and pests affecting the same host. Such an approach would allow multi‐disease protection while retaining the sequence specificity that distinguishes RNAi from conventional broad‐spectrum PPPs. Advances in bioinformatic target selection, off‐target prediction, and formulation technologies suggest that this strategy is technically feasible, yet it remains largely absent from current experimental pipelines.

### 6.2 Integrating dsRNA with peptide‐based plant protection strategies

A second underexplored direction lies in the integration of dsRNA‐based approaches with peptide‐based plant protection molecules. While dsRNA offers unparalleled specificity, its efficacy is constrained by delivery efficiency, environmental stability, and limited activity against certain organism groups, most notably bacterial pathogens.^106^ In contrast, antimicrobial peptides (AMPs) can effectively manage bacterial infections. Peptides are increasingly popular as novel plant protective molecules due to their antimicrobial properties and their role as stimulants. Notably, peptides have been shown to control insects, viruses, bacteria, oomycetes, and fungal pathogens affecting crops.^107^, ^108^, ^109^, ^110^ AMPs can be naturally derived or identified through the screening of large combinatorial libraries.^106^, ^110^ Their application in agriculture follows the same regulatory framework previously discussed for dsRNAs. AMPs are ideal environmentally compatible molecules that can be applied exogenously. We envision hybrid protection strategies in which peptides provide immediate or broad suppression, while dsRNA ensures sustained, sequence‐specific interference. Such combinations could reduce the required dose of each component, increase effectiveness of treatment, mitigate resistance development, and compensate for biological or environmental limitations inherent to each technology when used alone. In recent years, the use of peptide‐based bioinsecticides has expanded. An American company has introduced two peptide‐based bioinsecticides/acaricides containing the active ingredients GS‐omega‐Hxtx‐Hv1a, effective against thrips, whiteflies, aphids, and mites, and GS‐omega/kappa‐Hxtx‐Hv1a, targeting lepidopteran pests in field applications. Most recently, in 2024, the Greek and Italian Ministries of Agriculture granted emergency use approval for GS‐omega/kappa‐Hxtx‐Hv1a in both countries, marking an important step toward integrating peptide‐based biopesticides into mainstream agricultural practices in Europe.

## 7 TOWARD INTEGRATED AND NEXT‐GENERATION RNA INTERFERENCE‐BASED CROP PROTECTION

RNAi holds great promise as an innovative tool for plant disease management. Its potentially high specificity, environmental compatibility, and versatility make it an attractive addition to the current plant protection toolbox, particularly under increasing regulatory pressure and demand for sustainable practices. However, RNAi should not be regarded as a standalone solution. Its current limitations, including delivery robustness and the need for broader field‐level validation, highlight the importance of moving beyond one‐dimensional applications toward integrated and modular strategies.

RNAi technologies are currently transitioning from experimental validation to early‐stage commercialization, with practical deployment still confined to a limited number of crop‐target systems. Recent regulatory developments indicate that RNAi technologies are entering an initial commercialization phase. Recent regulatory approvals and submissions, particularly for insect control, demonstrate that translation from experimental research to formal evaluation is feasible under existing frameworks. However, adoption remains uneven across regions and targets, with significant variability in regulatory pathways, data requirements, and timelines. In Europe, dsRNA‐based products are still assessed within frameworks originally developed for chemical active substances, which may influence both the pace of authorization and investment decisions. Bridging this gap will require coordinated efforts to generate robust field data, strengthen environmental risk assessment, and optimize scalable and sustainable production and delivery platforms. Stronger collaboration between academia and industry is essential to accelerate target identification and validation, refine formulation strategies, and translate biological insight into reliable and deployable crop protection solutions.

RNAi should not be regarded solely as a replacement for traditional PPPs, but rather as a complementary tool to be integrated into disease management strategies. Recent evidence suggests that RNAi cannot only function independently but also enhance the efficacy of conventional fungicides when used in combination. In a study by Duanis‐Assaf et al.,^111^ the co‐application of dsRNAs targeting three essential transcripts active in the fungal ergosterol biosynthesis pathway of *B. cinerea* (dsRNA‐ERG) with the fungicide prochloraz (an ergosterol biosynthesis inhibitor) enabled a significant reduction in the fungicide dose. The combined treatment (1 mg L^−1^ prochloraz + dsRNA‐ERG) achieved the same inhibitory effect on fungal growth as a high concentration of prochloraz alone (1000 mg L^−1^), suggesting that RNAi can enhance the efficacy of existing PPPs and potentially reduce chemical input in crop protection. However, the same effect was not achieved with a different fungicide (fludioxonil, an inhibitor of MAP/Histidine‐Kinase in osmotic signal transduction). Therefore, the interaction between RNAi and PPPs may vary depending on the mode of action of the fungicide.

Integration within multifunctional platforms represents a promising direction for the future of RNAi‐based crop protection, where molecular specificity can be combined with other biotechnological solutions, such as AMPs to broaden activity range and enhance durability. In this context, RNAi is best positioned as a complementary component of integrated disease management strategies, capable of addressing pests and pathogens that remain difficult to control using conventional PPPs alone. RNAi represents a significant technological advancement, but like any agricultural innovation, it requires a balance between enthusiasm and caution. Experimental evidence has shown that sustained exposure to insecticidal dsRNA can select for resistant insect populations, as demonstrated in laboratory‐selected western corn rootworm and Colorado potato beetle strains, highlighting that RNAi‐based technologies, like other target‐specific control tools, may exert strong directional selection.^102^, ^112^ Therefore, resistance management strategies should be considered from the early stages of deployment.^113^ The challenge extends beyond technical and scientific aspects: achieving sustainability in crop protection with RNAi demands an integrated consideration of ecological, economic, social, and regulatory factors, avoiding ‘one‐dimensional’ solutions that risk neglecting indirect impacts or issues of accessibility. Social acceptance is essential and depends not only on transparent communication of risks and benefits but also on the active engagement of consumers, farmers, scientists, and policymakers to build trust and legitimacy. By embedding RNAi within holistic crop protection frameworks that account for ecological, social, and policy dimensions, RNAi‐based approaches could become a cornerstone of next‐generation, resilient, and sustainable agricultural systems.

## CONFLICT OF INTEREST

The authors declare no conflicts of interest in relation to this perspective and state that the opinions expressed are their own and should not be considered to reflect those of any other individuals or organizations.

## Data Availability

Data sharing not applicable to this article as no datasets were generated or analysed during the current study.
